# Genome-wide analysis and expression patterns of lipid phospholipid phospholipase gene family in *Brassica napus* L.

**DOI:** 10.1186/s12864-021-07862-1

**Published:** 2021-07-18

**Authors:** Wei Su, Ali Raza, Liu Zeng, Ang Gao, Yan Lv, Xiaoyu Ding, Yong Cheng, Xiling Zou

**Affiliations:** Oil Crops Research Institute, Key Laboratory of Biology and Genetic Improvement of Oil Crops, Chinese Academy of Agricultural Sciences (CAAS), Ministry of Agriculture, 430062 Wuhan, Hubei China

**Keywords:** Abiotic stress, Gene structure, Gene ontology, miRNA, Phytohormone, Lipid phosphate phosphatases, Rapeseed

## Abstract

**Background:**

Lipid phosphate phosphatases (LPP) are critical for regulating the production and degradation of phosphatidic acid (PA), an essential signaling molecule under stress conditions. Thus far, the *LPP* family genes have not been reported in rapeseed (*Brassica napus* L.).

**Results:**

In this study, a genome-wide analysis was carried out to identify *LPP* family genes in rapeseed that respond to different stress conditions. Eleven *BnLPPs* genes were identified in the rapeseed genome. Based on phylogenetic and synteny analysis, *BnLPPs* were classified into four groups (Group I-Group IV). Gene structure and conserved motif analysis showed that similar intron/exon and motifs patterns occur in the same group. By evaluating *cis*-elements in the promoters, we recognized six hormone- and seven stress-responsive elements. Further, six putative miRNAs were identified targeting three *BnLPP* genes. Gene ontology analysis disclosed that *BnLPP* genes were closely associated with phosphatase/hydrolase activity, membrane parts, phosphorus metabolic process, and dephosphorylation. The qRT-PCR based expression profiles of *BnLPP* genes varied in different tissues/organs. Likewise, several gene expression were significantly up-regulated under NaCl, PEG, cold, ABA, GA, IAA, and KT treatments.

**Conclusions:**

This is the first report to describe the comprehensive genome-wide analysis of the rapeseed *LPP* gene family. We identified different phytohormones and abiotic stress-associated genes that could help in enlightening the plant tolerance against phytohormones and abiotic stresses. The findings unlocked new gaps for the functional verification of the *BnLPP* gene family during stresses, leading to rapeseed improvement.

**Supplementary Information:**

The online version contains supplementary material available at 10.1186/s12864-021-07862-1.

## Background

Phospholipids exist in the cellular membranes of an organism. Most of them are structural, while a few serve as lipid-signaling molecules. Phosphatidic acid (PA) acts as a signaling compound and precursor for all phospholipids [1–3]. In plants, PA can be formed via three different pathways [1–3]. The PA abundance in plants is defined as the balance between enzymes responsible for PA synthesis and degradation. Phosphatidic acid kinase catalyzes PA phosphorylation to yield diacylglycerol pyrophosphate (DGPP). Phosphatidic acid phosphatase (PAP) is another key enzyme to keep a PA’s appropriate balance [4]. PAP can be divided into two types depending on the requirement of magnesium ion (Mg^2+^): (1) conventional PAPs, i.e., PAP1, the Mg^2+^-dependent PA phosphatase activities, and catalyzes PA dephosphorylation to generate diacylglycerol (DAG); and (2) PAP2, the Mg^2+^-independent PA phosphatase activities, named as lipid phosphate phosphatases (LPPs). These LPPs dephosphorylates PA to DAG but also dephosphorylates DGPP to PA [4]. In short, LPPs are members of the PAP superfamily and catalyze the dephosphorylation of phosphorous lipids, which play a vital role in numerous physiological functions, including cell migration, proliferation, and differentiation [3, 4].

Recently, significant progress has been made in the PAP superfamily. For instance, four PAP members (*APP1, DPP1, LPP1*, and *PAH1*) have been investigated in yeast. Where *APP1* and *PAH1* are Mg^2+^-dependent, and *DPP1* and *LPP1* are Mg^2+^-independent PAPs. Notably, *PAH1* is the major regulator of triacylglycerol(s) (TAG) content [5, 6], and *DPP1* and *LPP1* play a crucial role in controlling the signal transduction of PA, DAG, and DGPP [7–9]. Plants also contain multiple PAP isoforms such as *PAP1* (*PAH1* and *PAH2*), similar to yeast *PAH1*, PAPs responsible for galactolipid synthesis [10], and transiently increased the PA and DGPP synthesis under multiple stresses in plants. In agreement, LPPs were found to be responsible for switching these signals on/off under stress conditions [11]. The LPP-mediated DAG production significantly affects the invasion and growth of *Magnaporthe oryzae* [5, 12]. In another study, four *PAP2/LPP* genes were cloned in *Arabidopsis thaliana*, similar to yeast *LPPs* [4, 13]. Northern blot analysis revealed that *AtLPP1* was more likely to be expressed in leaves and roots, while the expression of *AtLPP2* was recognized in all the tested tissues of *A. thaliana* [13]. Genotoxic (gamma-ray or UV-B) and elicitor treatments transiently induced the *AtLPP1* and *AtLPP2* expression levels involved in abscisic acid (ABA) signal transduction and stomatal movement [14, 15]. Physiological analysis showed that PA accumulation triggers early signal transduction actions that lead to ABA responses during seed germination and regulate the stomatal movement [14, 15]. Interestingly, PA is involved in ABA signaling, and thus *AtLPP2* also serves as negative regulators in ABA-induced seed germination inhibition [15]. The *HvLPP1/2* genes are involved in ABA sensitivity and breaking dormancy in barley (*Hordeum vulgare* L.) [16]. According to the literature, LPPs enzymes are involved in lipid synthesis and thus regulate plants’ growth. For example, *VuPAPa* and *VuPAPb* may be involved in membrane lipid modification, observed in cowpea (*Vigna unguiculata* L.) plants under drought stress [17]. The knock-down of *NtLPP4* inhibited PA degradation and promoted pollen tube growth in tobacco (*Nicotiana tabacum*) plants [18].

Rapeseed (*Brassica napus* L.) is considered the second most important oilseed crop and serves as a primary oil source for human consumption and animal feed meals [19]. Numerous environmental stresses adversely affect rapeseed growth, productivity, and seed quality, ultimately reducing the final yield [19]. To date, *LPP* family genes are yet to be reported in rapeseed. The complete rapeseed genome sequence allows the identification and analysis of *LPP* genes in the rapeseed genome. Hence, a genome-wide comprehensive study has been performed to identify putative rapeseed *LPP* family genes. Additionally, their phylogenetic relationships, synteny analysis, gene structures, conserved motifs, *cis*-elements, miRNA regulator prediction, functional annotation have been characterized to get insights into the *BnLPP* genes. Moreover, the expression profiles in different tissues/organs and under numerous hormone and abiotic stresses have been extensively assessed.

## Results

### Identification and characterization of *LPP* gene family in *Brassica napus* L

In the current study, 11 *BnLPPs* genes were obtained containing the complete PAP2 functional domain (Table 1). Six genes were positioned in the A subgenome, and five genes were positioned in the C subgenome (Table 1). Detailed characteristics of 11 *BnLPP* genes are presented in Table 1. Briefly, coding DNA sequences (CDS) length ranged from 918 to 1089 bp with 2–8 exons, and the protein length ranged from 305 to 362 amino acids for *BnLPP2A*/*BnLPP4A*/*BnLPP4B*, and *BnLPP3A*/*BnLPP3B*, respectively. The protein molecular weight (MW) ranged from 34.7 kDa (*BnLPP4A* and *BnLPP4B*) to 40.5 kDa (*BnLPP3A*), and isoelectric points (pI) varied from 6.13 (*BnLPP2A*) to 8.64 (*BnLPP1B*). The subcellular location prediction revealed that 10 BnLPP proteins were positioned in the plasma membrane, while *BnLPP1C* was located in the endoplasmic reticulum. Meanwhile, 4 *Brassica oleracea* (*BoLPP1A-BoLPP4*), 6 *Brassica rapa* (*BraLPP3B-BraLPP2B*), and 4 *Arabidopsis thaliana* (*AtLPP1-AtLPP4*) LPP genes were also identified (Additional file 2).

**Table 1:** The characteristics of 11 *BnLPPs* in *Brassica napus* L

Gene name	Gene ID	Genomic position (bp)	CDS length (bp)	Exon	Protein length (aa)	MW	pI	Predicted Pfam domain	Subcellular location
*BnLPP1A*	BnaA09g18500D	A09:11,485,093–11,486,357 -	984	2	327	36.9	8.35	PAP2	PM
*BnLPP1B*	BnaC09g20440D	C09:17,421,172–17,423,263 -	972	2	323	36.6	8.64	PAP2	PM
*BnLPP1C*	BnaA06g35100D	A06:23,178,693–23,179,882 +	951	2	316	35.5	7.20	PAP2	ER
*BnLPP2A*	BnaC08g39060D	C08:34,977,648–34,979,784 -	918	7	305	35.6	6.13	PAP2	PM
*BnLPP2B*	BnaA09g45250D	A09:30,966,399–30,968,509 -	939	7	312	35.2	6.18	PAP2	PM
*BnLPP3A*	BnaC05g48240D	C05:42,805,948–42,809,057 +	1089	8	362	40.5	6.13	PAP2	PM
*BnLPP3B*	BnaA05g33490D	A05:22,673,065–22,676,351 +	1089	8	362	40.4	6.23	PAP2	PM
*BnLPP3C*	BnaC03g33070D	C03:20,223,052–20,225,854 +	966	6	321	36.1	6.53	PAP2	PM
*BnLPP3D*	BnaA03g28040D	A03:13,723,633–13,726,024 +	966	6	321	36.1	6.56	PAP2	PM
*BnLPP4A*	BnaA05g21920D	A05:16,840,547–16,842,185 -	918	6	305	34.7	8.46	PAP2	PM
*BnLPP4B*	BnaC05g35130D	C05:34,426,978–34,428,721 -	918	6	305	34.7	8.46	PAP2	PM

In the genomic position, the positive (+) and negative (-) sign indicates the existence of gene on the positive and negative strand of that specific markers, respectively

*CDS* coding DNA sequences, *bp* base pair, *MW* molecular weight, *pI* isoelectric points, *PM* plasma membrane, *ER* endoplasmic reticulum

### Multiple sequence alignment and phylogenetic analysis of *BnLPP* gene family

**Fig. 1:**
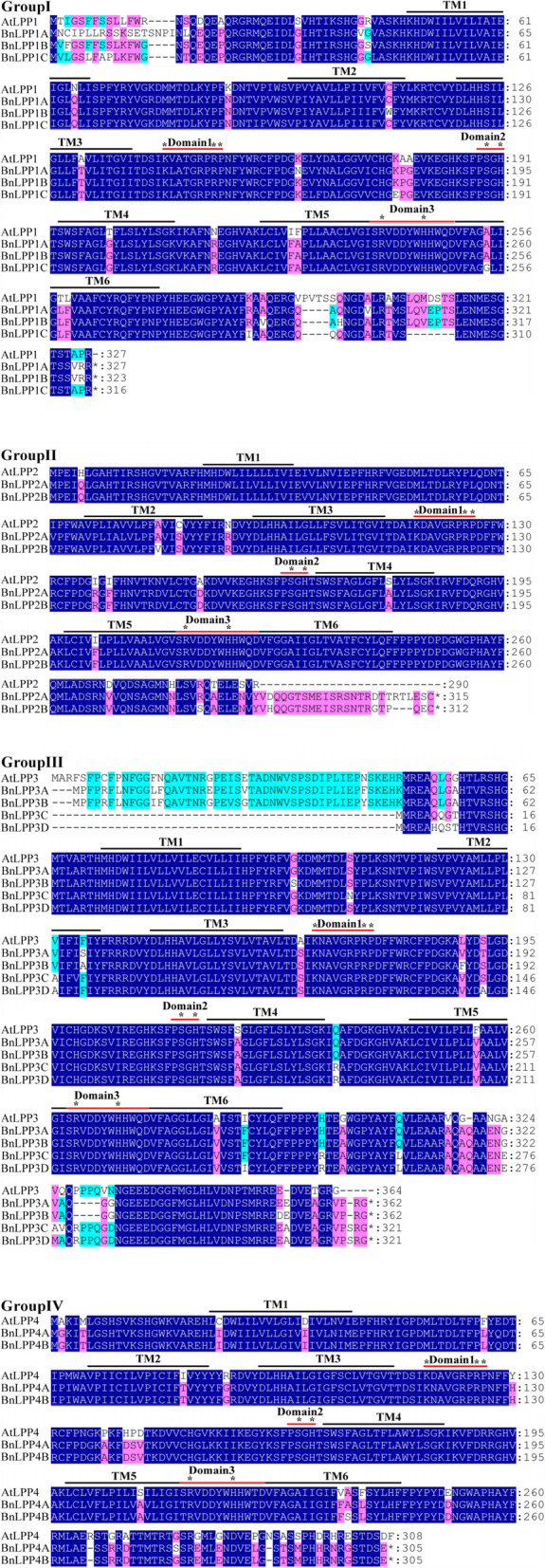
Alignment of multiple BnLPPs and selected AtLPPs protein sequences

To understand the sequence characteristics, we performed a multiple sequence alignment analysis of the 11 BnLPP proteins using DNAMAN software with the default parameters. The four different *A. thaliana* LPP proteins (AtLPP1-AtLPP4) from each group were randomly selected as representatives for further comparison. The transmembrane structure and conversed domain structures of BnLPPs are displayed in Fig. 1. It was predicted that all the LPP proteins contained six membrane-spanning hydrophobic regions, named TM1-6 by TMHMM [19]. Our results showed that the PAP2 domains were highly conserved and commonly contained three consensus domains (denoted by a red bar), i.e., KX_6_RP (domain 1), PSGH (domain 2), and SRX_5_HX_3_D (domain 3). Notably, the conserved amino acids in the PAP2 domain were found to be essential for enzymatic activity. Thus, alteration in these amino acids may cause severe gene function losses [20].

**Fig. 2:**
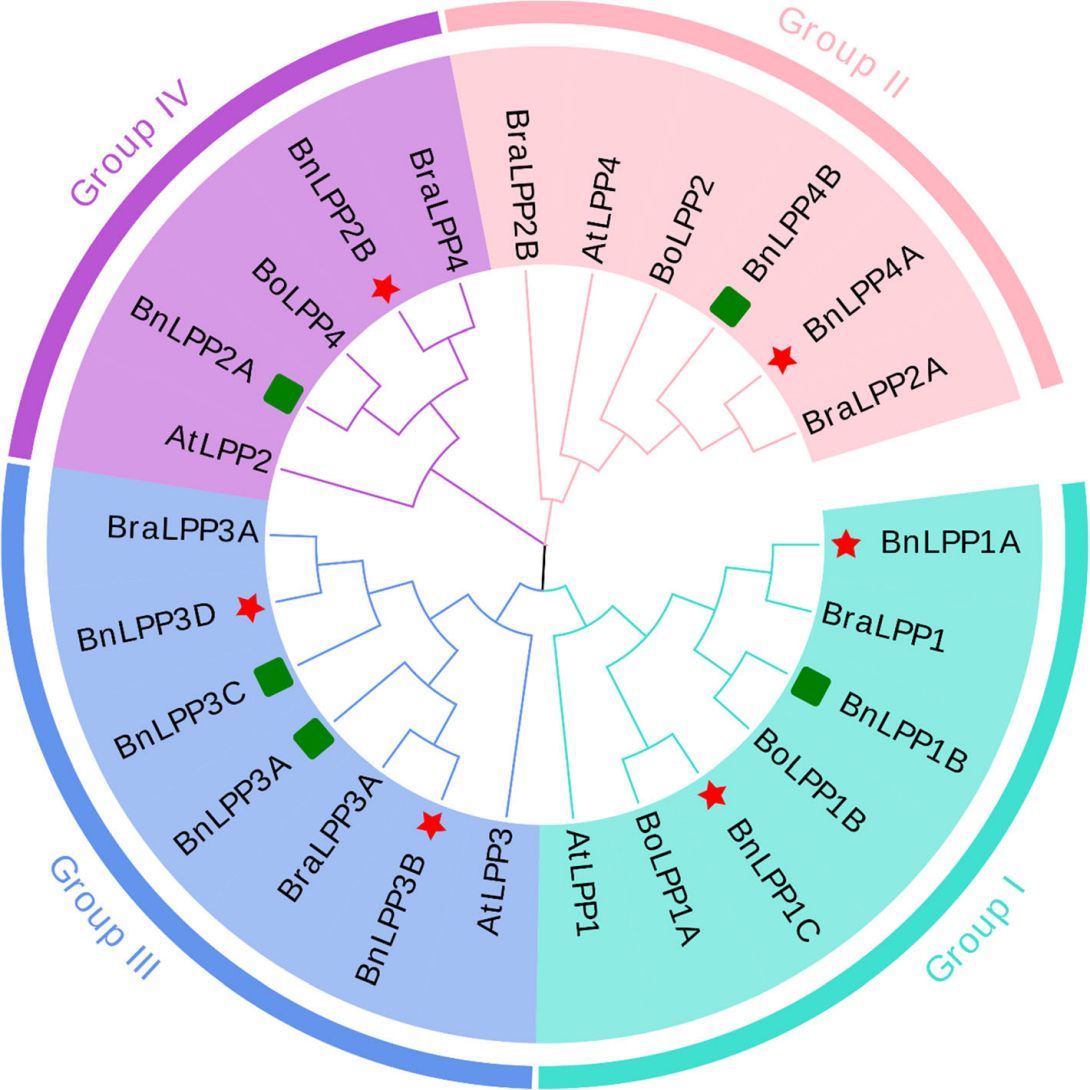
A phylogenetic tree of 25 *LPPs* from *B. napus, B. oleracea, B. rapa*, and *A. thaliana*. All *LPPs* genes were divided into four groups based on the high bootstrap values and the phylogenetic tree’s topology. Overall, 11 *BnLPPs* from *B. napus*, 6 *BraLPPs* from *B. rapa*, 4 *BoLPPs* from *B. oleracea*, and 4 *AtLPPs* from *A. thaliana* were clustered into four groups (Group I-IV) based on high bootstrap values signified with different background colors. The red star and green rectangle indicate that these genes belong to the A and C subgenome, respectively

To determine the *BnLPP* genes family’s evolutionary relationships with *(A) thaliana* and the *(B) napus* ancestor species, based on the neighbor-joining (NJ) method, an unrooted phylogenetic tree was constructed between 25 LPP genes (11 from *B. napus*, 6 from *B. rapa*, 4 from *B. oleracea* and 4 from *(A) thaliana*). The phylogenetic analysis indicated that the 25 LPPs were grouped into four groups (Group I, II, III, and IV) (Fig. 2). Our results showed that Group I contained 7 *LPPs* members (3 *BnLPPs*, 1 *BraLPP*, 2 *BoLPPs*, and 1 *AtLPP*), Group II contained 6 LPPs members (2 *BnLPPs*, 2 *BraLPPs*, 1 *BoLPP*, and 1 *AtLPP*), Group III contained 7 LPPs members (4 *BnLPPs*, 2 *BraLPPs*, and 1 *AtLPP*), and Group IV contained 5 *LPPs* members (2 *BnLPPs*, 1 *BraLPP*, 1 *BoLPP*, and 1 *AtLPP*) (Fig. 2). Overall, *LPPs* grouping into the same sub-group may have similar functions. Notably, all LPPs members were evenly distributed in four groups; however, no *BoLPPs* belonged to Group III (Fig. 2). Moreover, it was found that the *BnLPPs* have close phylogenetic relationships with their ancestors’ species in each group. *Arabidopsis* and *Brassicas* have a common ancestor, but *AtLPP3* had no *(B) oleracea* homologous gene in Group III, indicating that a few genes were lost during the *Brassica* species’ evolution.

In the aligned amino acids, invariant ones were marked with black, and the conserved ones were marked with blue (3/3, 4/4, and 5/5), purple (2/3, 3/4, and 4/5), and cyan (2/4 and 3/5). Black bars represented the six transmembrane regions, and red bars represented the three domains of the phosphatase motif. Asterisks represented the conserved amino acid residues.

### Gene structure and conserved motif composition of *BnLPPs* gene family

**Fig. 3:**
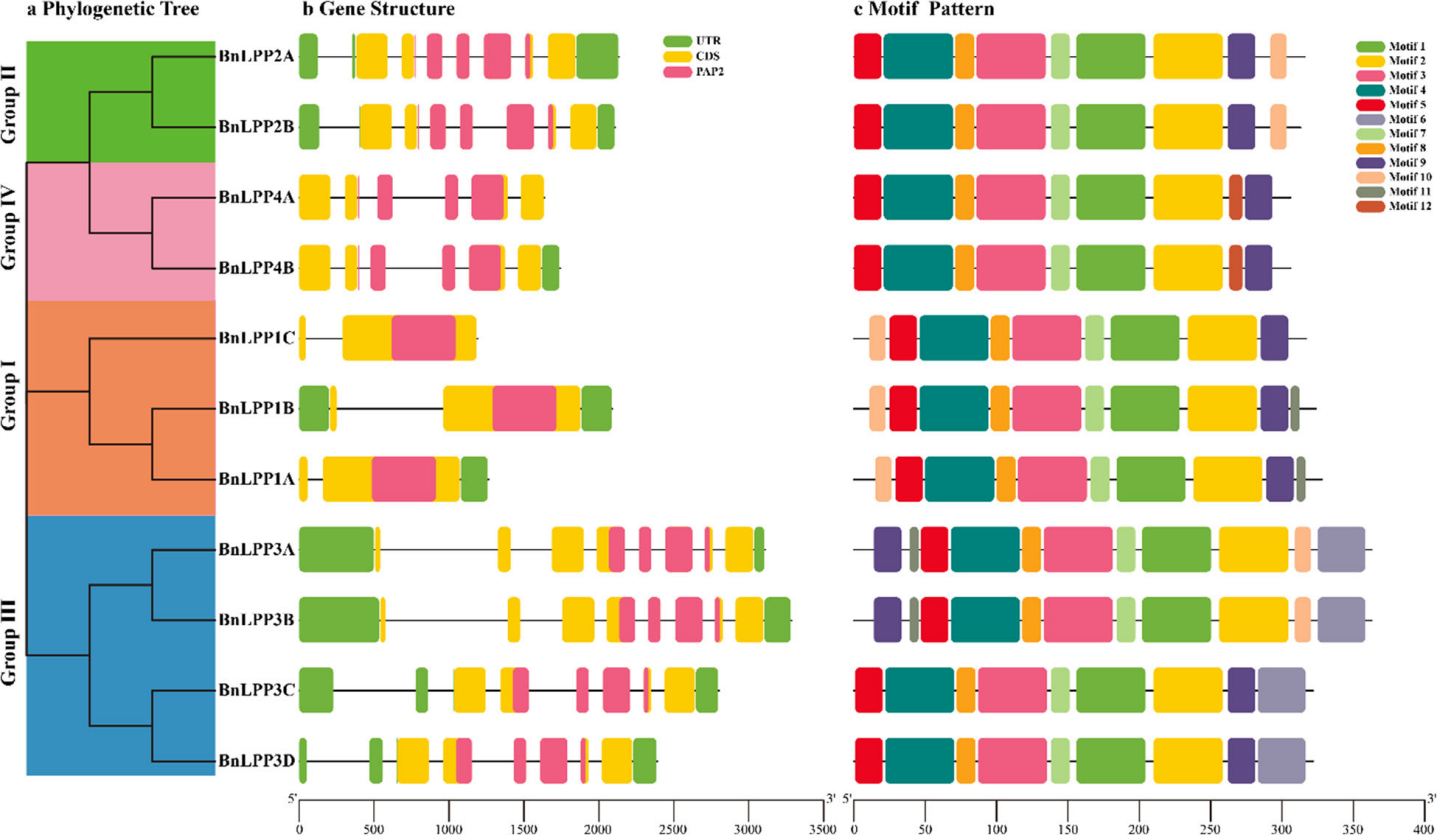
Phylogenetic relationships, gene structure, and architecture of conserved protein motifs in *BnLPPs*. **a** A phylogenetic tree based on the *BnLPPs* sequences. According to phylogenetic relationships, 11 *BnLPPs* were clustered into four groups (I-IV) and represented with different colors. **b** The exon-intron structure of *BnLPPs*. Green boxes indicate UTR regions, yellow boxes indicate exons, blackish-grey lines indicate introns, and pink boxes indicate PAP2 domain. **c** The motif composition of *BnLPPs*. Different colored boxes display different motifs. The details of each motif were presented in Additional file 3. The bottom scale shows the protein length

The exon-intron configurations of *BnLPPs* genes were examined to acquire further insights into the probable structural evolution of *BnLPP* family genes. Our results display that the number of exons of *BnLPPs* ranged from 2 to 8 (Table 1; Fig. 3). We also found that similar structures usually exist in the same group, e.g., the group I members have one intron and two exons. Likewise, groups II, III, and IV contained three or four introns in their respective PAP2 domains except for group I (Fig. 3a and b). Mainly, groups II and IV had a diverse intron/exon association pattern. These results showed that members within a group had a similar intron/exon pattern, consistent with the clusters of *BnLPPs*.

Furthermore, we investigated the full-length protein sequences of 11 *BnLPPs* to recognize their conserved motifs. Generally, 12 conserved motifs were identified, and motifs 1, 2, 3, 4, 5, 7, and 8 were found to be widely distributed. Interestingly, *BnLPPs* in the same group tends to have similar motif composition (Fig. 3c). For example, motif 12 was specific to group IV, while motif 6 was specific to group III (Fig. 3a). The similar motif arrangements in subgroups indicated the protein structure was conserved within a specific subfamily. Overall, the results reveal that members inside a group had identical gene structures, constant with their phylogenetic relationships. The group classifications’ stability was convincingly maintained by studying conserved motif compositions, gene structures, and phylogenetic relationships, showing that BnLPP proteins have very conserved amino acid residues, and members within the group may have analogous functions.

### Chromosomal distribution and synteny analysis of *BnLPP* genes

**Fig. 4:**
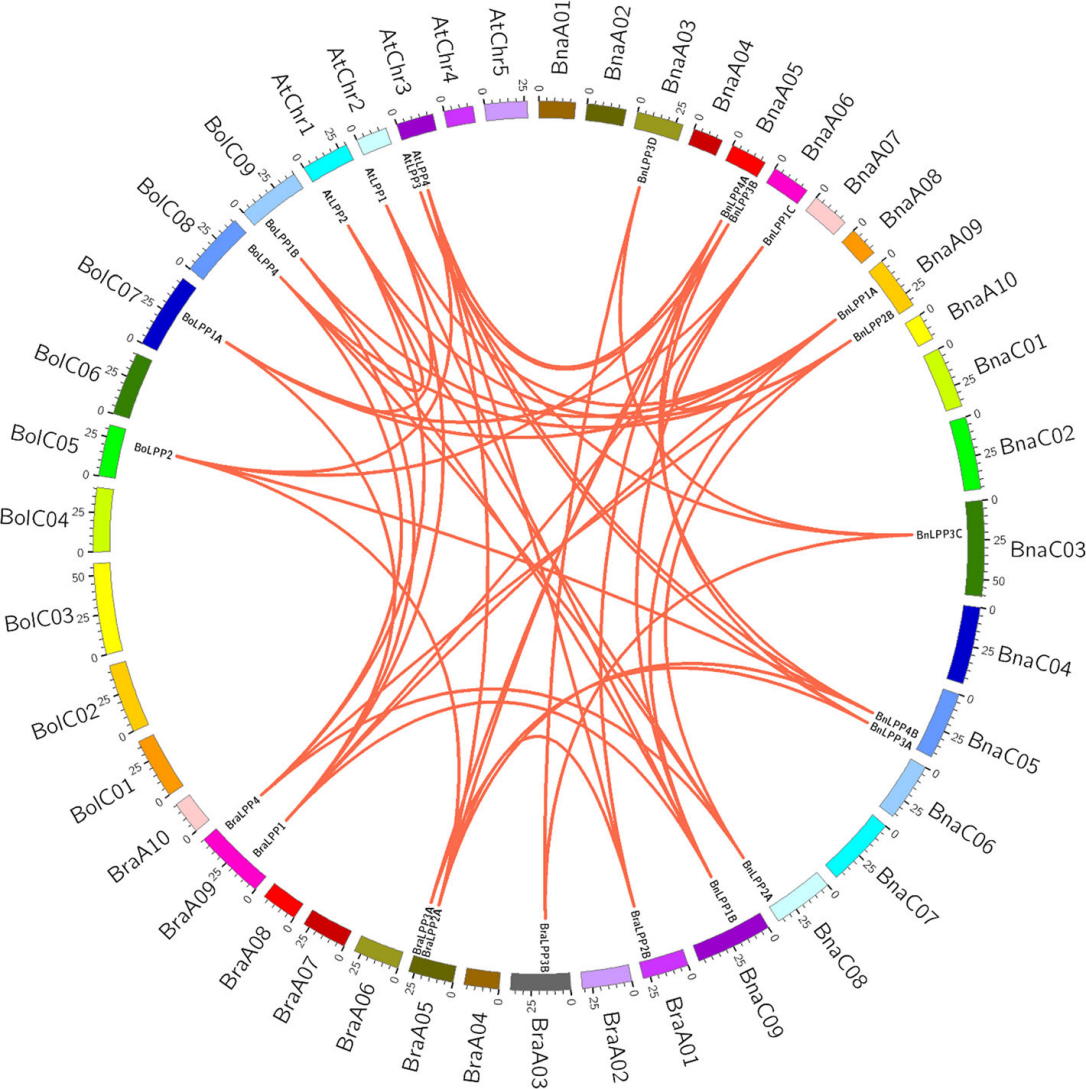
Synteny analysis of LPPs in *A. thaliana*, *B. rapa*, *B. olerecea*, and *B. napus*. The red lines represented the syntenic LPP pairs between the two genomes. The chromosome number was shown at the bottom of each chromosome

The expansion of new gene family members in plant genome evolution is partly attributed to tandem and segmental duplication [21], and the corresponding events were studied in *BnLPPs*. The chromosomal location of 11 *BnLPPs* was evaluated, and the result shows that 8 out of the 19 chromosomes had *BnLPP* genes (Table 1). Briefly, chromosomes A05, A09, and C05 harbored 2 *BnLPPs*, whereas other chromosomes (A03, A06, C03, C08, and C09) possess only one *BnLPP* gene (Table 1). However, despite A05 and C05 possess gene clusters (*BnLPP4A* and *BnLPP3B*, and *BnLPP4B* and *BnLPP3A*), no tandem duplication events were found in these regions (Fig. 4; Additional file 4). Additionally, we also identified 6 and 4 *LPPs* genes in the *B. rapa* and *B. oleracea* genomes, respectively (Additional file 2). Our findings show that these genes were similar to those in the A and C sub-genomes of *B. napus*.

Collinearity analysis revealed orthologs (speciation events) among the *B. napus*, *B. rapa*, *B. oleracea*, and *A. thaliana LPP* genes (Fig. 4). There was a tripling in *Brassica* species after diversion from their common ancestor with *(A) thaliana* [21]. Therefore, one *AtLPP* should theoretically correspond to three orthologs in *(B) rapa* and *B. oleracea*. However, more than one homologous gene of *AtLPP1*, *AtLPP2*, and *AtLPP4* in *B. rapa* and *B. oleracea* and two-four homologous genes in the *B. napus* genomes (in both A and C subgenome) have been predicted in different groups (Fig. 2; Additional file 4). Interestingly, *AtLPP3* has no homologous genes in *B. oleracea*, but four homologous genes in *B. napus*, located on A- (2) and C-subgenome (2) (Additional file 4). The synteny between *BraLPPs, BoLPPs*, and *AtLPPs* homologs genes was less than expected (4:6:4), indicating that duplicated genes might have been lost during evolution. Additionally, all *BnLPPs* genes were found to be associated with twelve and eight syntenic gene pairs, particularly between *B. rapa* and *B. oleracea LPP* genes. These results indicate that allotetraploidy was the main force for the rapid expansion of the *LPP* gene family in *B. napus*. Moreover, all *LPP* genes were obtained by whole-genome duplication (WGD; polyploidy) and segmental duplication events, and there was no putative tandem duplication. Overall, our results indicate that the *LPP* gene family’s expansion in the *B. napus* genome was mainly due to WGD and segmental duplication.

The ratio of Ka and Ks is an important index to evaluate repeated events’ positive selection pressure [21, 22]. The Ka/Ks of duplication *BnLPPs* varied from 0.0707 to 0.1712, and the mean value was 0.1012. All the duplicated *BnLPPs* gene pairs had the Ka/Ks values were less than 1 (Additional file 5), suggesting a strong purifying selective pressure occurred during the evolution of *BnLPPs*.

### *Cis*-Elements in the promoters of *BnLPPs*

**Fig. 5:**
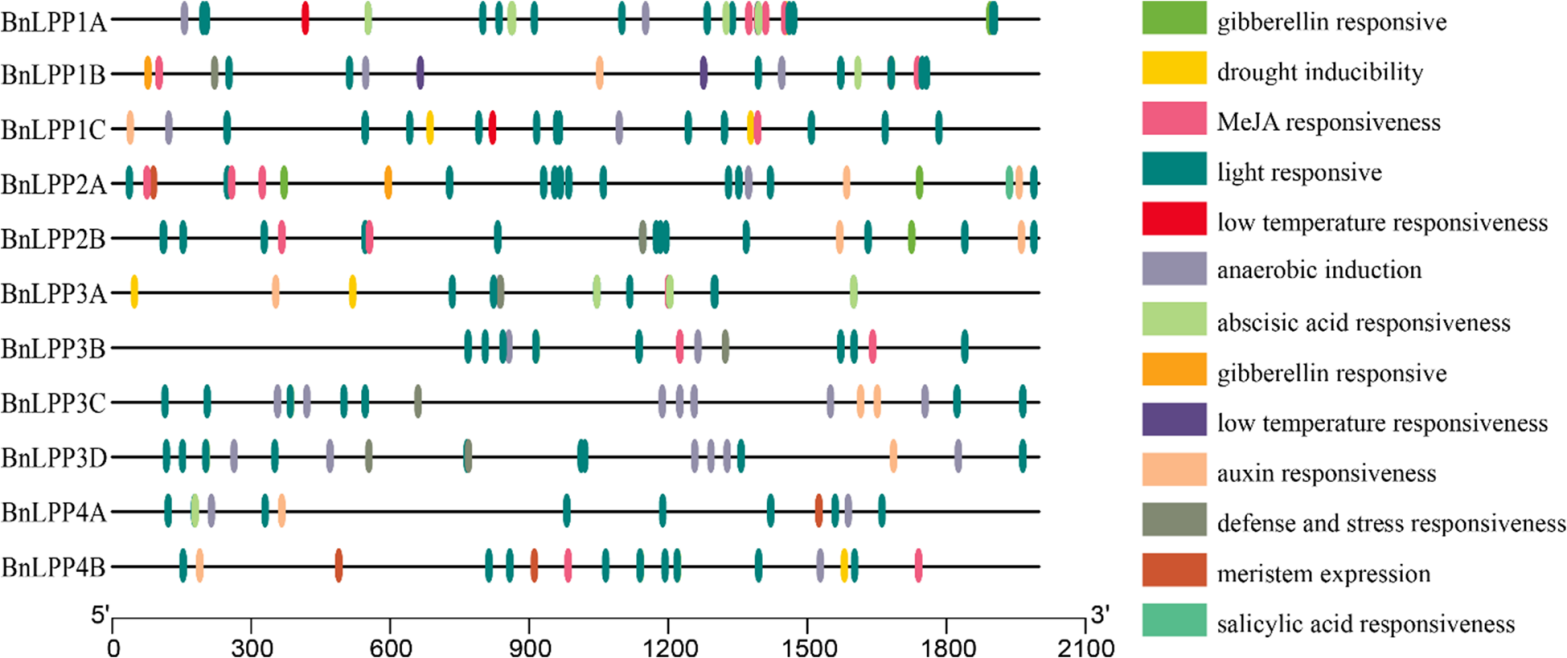
*Cis*-elements that are related to different stress and hormone responses in the putative promoters of *BnLPPs*. *Cis*-elements with similar functions were displayed in the same color. Different color boxes show different identified *cis*-elements

In order to explore gene function and regulation patterns, we studied the *cis-*elements in the region of 2000 bp upstream of the initiation codon of each *BnLPPs*. Our results revealed three major classes of *cis*-elements, i.e., stress-, hormone-, and light-responsive elements. Overall, 13 putative *cis*-elements were predicted in the *BnLPPs* promoter (Fig. 5). Among them, six hormone-responsive [(abscisic acid (ABA), auxin, methyl jasmonate (MeJA), gibberellin (GA), and salicylic acid (SA)], and remaining were associated with drought stress, low-temperature stress, defense, anaerobic induction, and meristem expression (Fig. 5). Relatively more light-responsive *cis*-elements were observed in the *BnLPPs* promoters (Additional file 6). As shown in Fig. 5, most of the hormone- and stress-responsive elements were specific to some genes highlighting their crucial role in hormone and stress response mechanisms.

### Functional annotation analysis of *BnLPP* genes

To further discriminate the *BnLPP* genes’ functions, we implemented gene ontology (GO) annotation and enrichment analysis based on three classes, i.e., biological process (BP), molecular function (MF), and cellular component (CC). These GO terms boost our understanding of the precise gene functions. The GO annotation outcomes revealed numerous significantly enriched terms (Additional file 7). For instance, the GO-BP enrichment results revealed seven enriched terms, including cellular process (GO:0009987), phosphorus metabolic process (GO:0006793), phosphate-containing compound metabolic process (GO:0006796), dephosphorylation (GO:0016311), etc. (Additional file 7). The GO-CC enrichment outcomes discovered 13 enriched terms such as obsolete membrane part (GO:0044425), cell periphery (GO:0071944), an integral component of membrane (GO:0016021), obsolete plasma membrane part (GO:0044459), etc. (Additional file 7). Nearly all GO-CC terms are consistent with the subcellular localization of the BnLPP proteins. Likewise, GO-MF enrichment findings exposed eight enriched terms, including phosphatidate phosphatase activity (GO:0008195), phosphoric ester hydrolase activity (GO:0042578), phosphatase activity (GO:0016791), catalytic activity (GO:0003824), etc. (Additional file 7). In short, GO enrichment outcomes validate the functional role of *BnLPP* genes in numerous biological, cellular, and molecular processes that were associated with phosphatase activity, hydrolase activity, membrane parts, phosphorus metabolic process, and dephosphorylation.

### Genome-wide analysis of miRNA targeting *BnLPP* genes

**Fig. 6:**
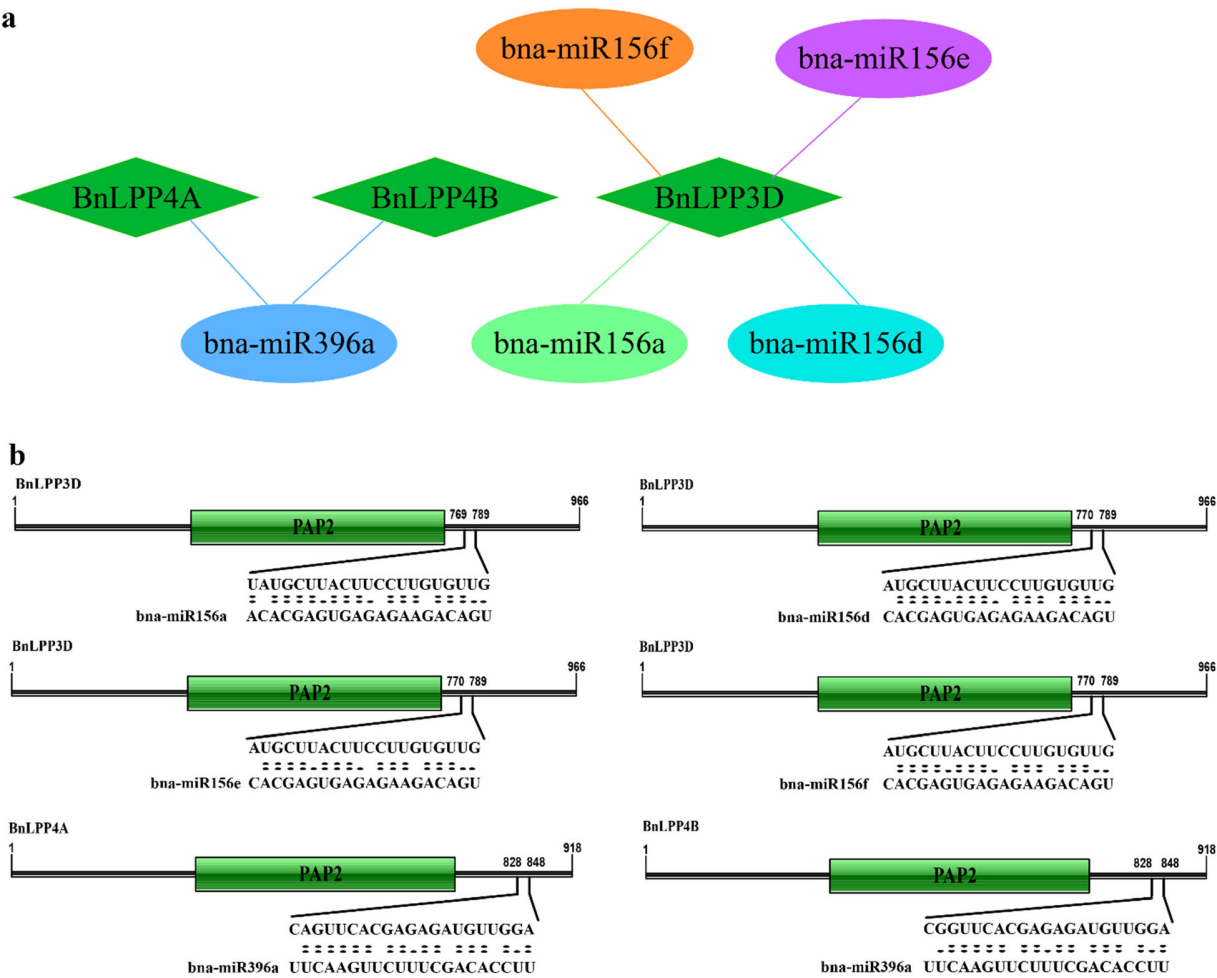
**a** A network diagram of the regulatory relationships between the presumed miRNAs and particular *BnLPP* genes. **b** Prediction of miRNA target sites. Thick black lines represent the CDS of *BnLPPs*. PAP2 domain is shown in green boxes. miRNA complementary sites (black filling) with the nucleotide positions of *BnLPPs* are specified

In recent years, numerous researchers have discovered that microRNA (miRNA)-mediated regulation is accompanying plants’ stress responses. Thus, to increase our knowledge of miRNAs connected with *BnLPP* gene regulation, we identified six putative miRNAs targeting three *BnLPP* genes (Fig. 6a). Prediction of miRNAs target sites is illustrated in Fig. 6b and Additional file 8. Our results showed that four members of the bna-miR156 family targeted one gene (*BnLPP3D*), and two members of the bna-miR396 family targeted two genes (*BnLPP4A* and *BnLPP4B*) (Fig. 6; Additional file 8). Predominantly, *BnLPP3D* was targeted by four miRNAs (bna-miR156a, bna-miR156d, bna-miR156e, and bna-miR156f).

### Expression patterns of *BnLPP*s in different tissues

**Fig. 7:**
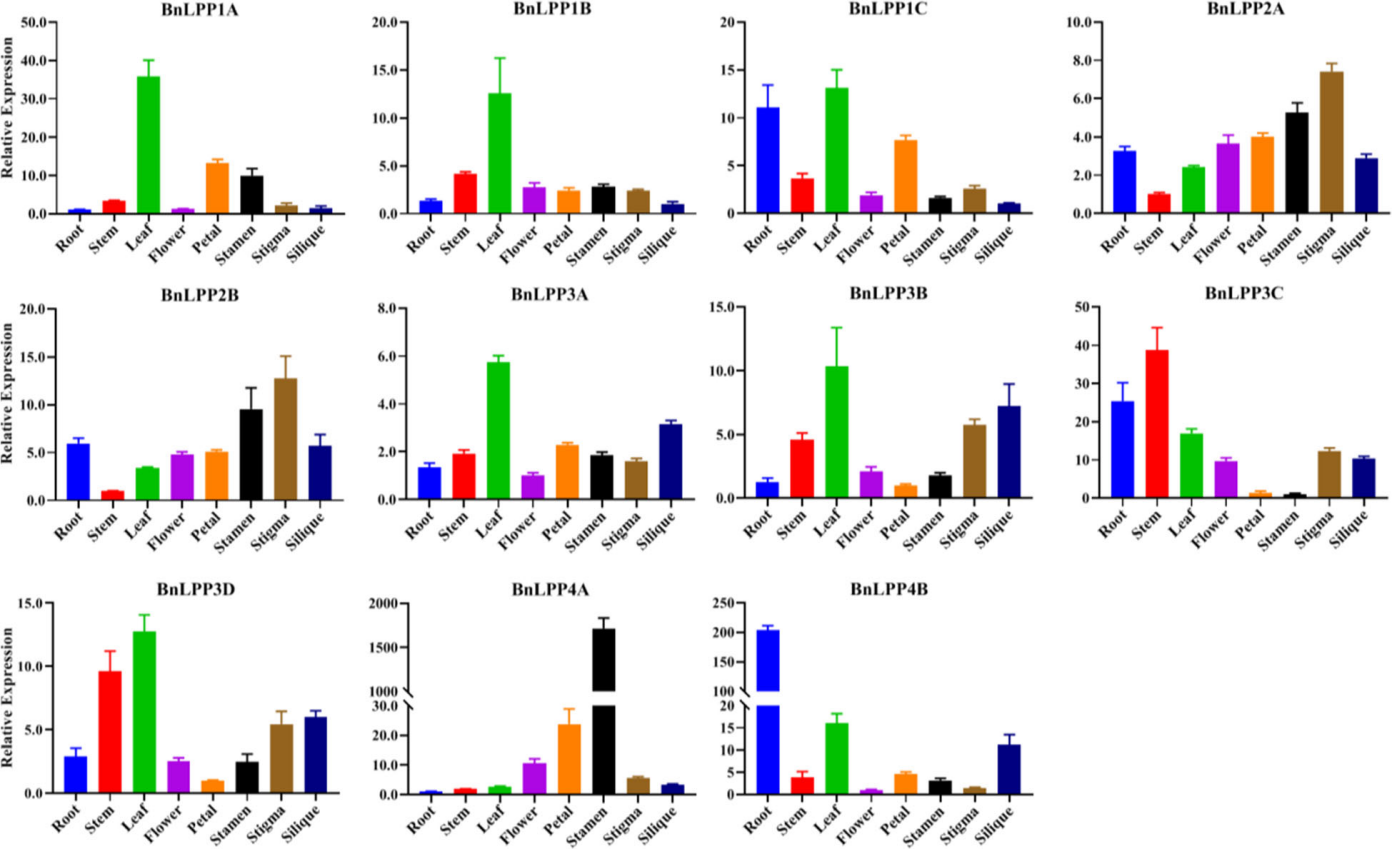
*BnLPPs* expression profilesin differenttissues. The data were normalized to*β-actin*,and the standard deviation was represented by vertical bars

To demonstrate the expression patterns of the *BnLPP* genes, we examined eight tissues/organs (roots, stem, leaves, flower, petal, stamen, stigma, and silique) of rapeseed at various growth phases by qRT-PCR (Fig. 7). The expression profiles of *BnLPP* genes varied in the various tissues/organs. For instance, the expression of 6 genes (*BnLPP1A*, *BnLPP1B*, *BnLPP1C*, *BnLPP3A*, *BnLPP3B*, and *BnLPP3D*) were higher in leaves compared to other tissues (Fig. 7). The expression levels of *BnLPP2A* and *BnLPP2B* were higher in stigma. The expression of *BnLPP4A* was higher in stamens. The expression level of *BnLPP4B* was higher in roots, and *BnLPP3C* showed higher expression in stems. Our results advise that these candidate genes may play dynamic roles in advancing *B. napus* developmental processes.

### Expression patterns of *BnLPP* genes under different stress and phytohormone treatments

**Fig. 8:**
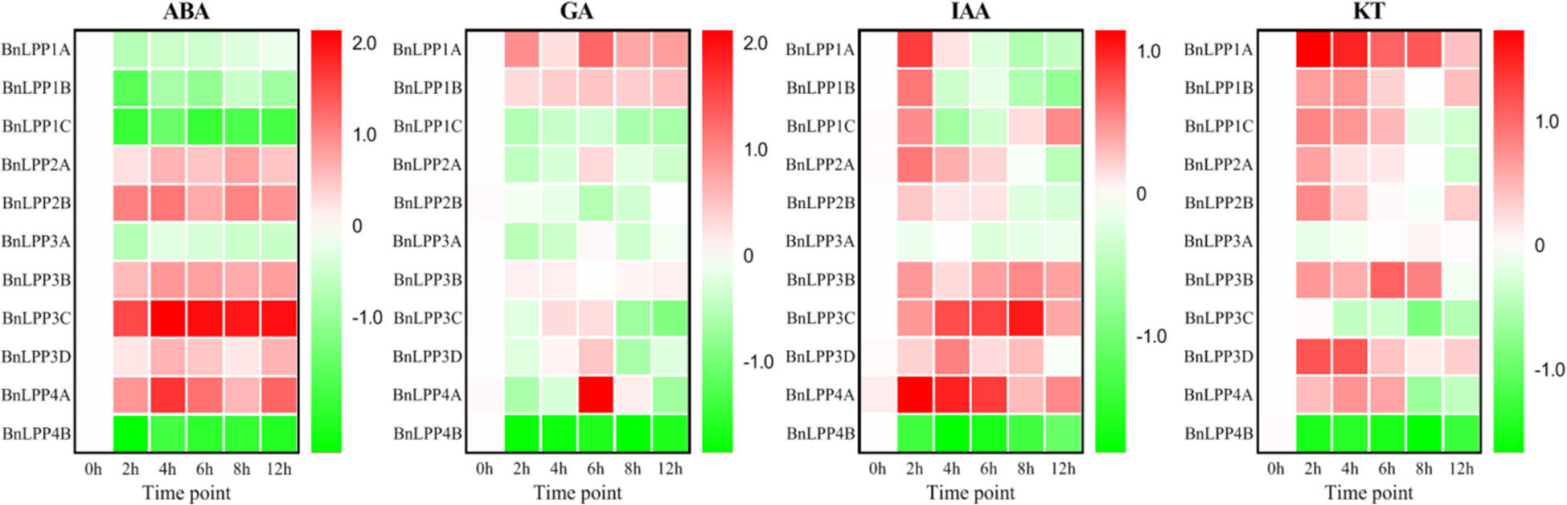
Expression profiles of 11 *BnLPPs* under abiotic stress.The datawerenormalized to *β-actin*. The heatmap showed the change ratio of the *BnLPPs*expression levels under NaCl, cold,and PEG stress. The red color displays high, and the green color displays low expression levels

**Fig. 9:**
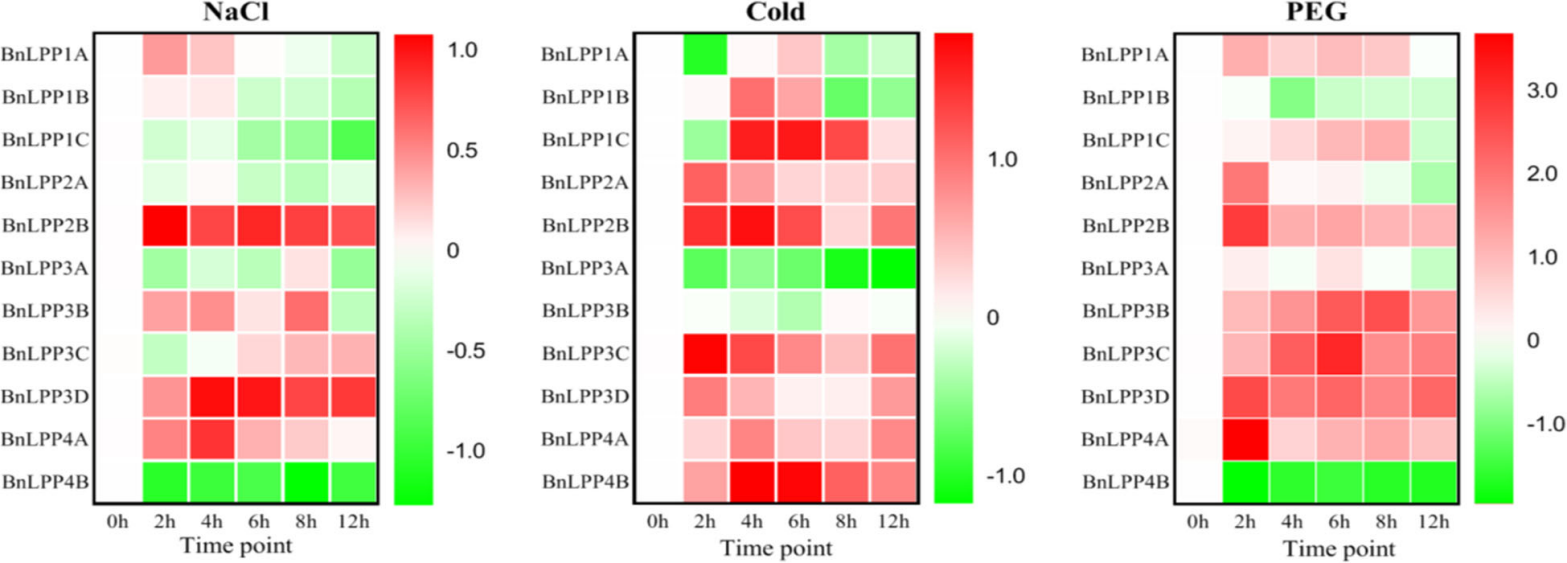
Expression profiles of 11 *BnLPP* genes in response to different hormones. The data were normalized to the expression of *β-actin*. The heatmap showed the change ratio of the *BnLPPs* expression levels under ABA, GA, IAA, and KT. The red color displays high, and the green color displays low expression levels

Under stress conditions, *B. napus* growth and developmental process greatly influenced physiological, biochemical, and molecular mechanisms. Hence, qRT-PCR was used to examine 11 *BnLPP* genes’ expression patterns at different time points after PEG, NaCl, and cold (Fig. 8), ABA, GA, IAA, and KT treatments (Fig. 9). In general, the expression of some *BnLPPs* was significantly induced by various treatments. For example, *BnLPP1A* was significantly responsive to NaCl, GA, IAA, and KT treatments. All treatments except GA induced the expression of *BnLPP2B*. Meantime, most *BnLPPs* (*BnLPP1A/2B/4A/3B/3C/3D*) were induced by one specific treatment. For instance, six *BnLPPs *(*BnLPP1A/2B/4A/3B/3C/3D*) were significantly up-regulated by NaCl treatment, and nine genes (*BnLPP1B/1C/2A/2B/4A/4B/3B/3C/3D*) were up-regulated by cold stress. Salt stress decreased the expression levels of *BnLPP1C* and *BnLPP4B*. Several genes indicated opposite expression patternsunder different hormone conditions. For instance,* BnLPP1C* was significantly increased by IAA or KT and inhibited by ABA or GA treatments. In short, results suggest that these genes may be vital for enlightening tolerance to numerous stresses, i.e., hormone and abiotic stress conditions.

## Discussion

Rapeseed is an allotetraploid that practiced widespread genome repetition and integration events [23]. Nevertheless, rapeseed yield is affected by numerous environmental cues [19]. LPP plays a vital role in catalyzing phosphorous lipids’ dephosphorylation, which plays an active part in several physiological processes, including cell migration, proliferation, and differentiation [3, 4]. So far, only a few numbers of *LPP* genes have been recognized in plants. A genome-wide analysis is a crucial method for clarifying the biological functions of the *LPP* family associates in a particular plant species. Over the past few years, *LPP* family genes have been examined in different plant species, such as *A. thaliana* [13–15], barley [16], cowpea [17], and tobacco [18]. So far, there is no comprehensive study of the *LPP* gene family in rapeseed. The accessibility of the whole rapeseed genome permits the genome-wide characterization of the *LPP* family genes, which may further be used for rapeseed improvement.

In the present study, a total of 11 *BnLPP* genes (six on A- and five on C-subgenome) were identified in the rapeseed genome (Table 1), which surpasses the number of genes identified from closely related species (4 *BoLPPs*, 6 *BraLPPs*, and 4 *AtLPPs* genes) (Additional file 2). This may be due to the rapeseed genome duplication (A and C subgenomes). Notably, *AtLPP2* and *AtLPP4* had only one corresponding *B. rapa* and *B. oleracea* orthologous gene. However, *AtLPP3* had no orthologous in *B. oleracea*, and two *AtLPP3* orthologous were determined in *B. rapa*. Further, the *B. oleracea* was compared with its sister specie, i.e., *B. rapa* to reveal numerous chromosome rearrangements and asymmetrical gene loss in duplicated genomic blocks. The results show that the relative rates of gene loss/retention were consistent with asymmetrical evolution between *B. oleracea, B. rapa*, and *(A) thaliana* genomes. This evidence supports the role of asymmetrical evolution in the evolutionary success of polyploidy as well as their phenotypic novelty and adaptation [24, 25]. Further, the results indicated that most duplicated *LPP* genes were lost after *Brassica* genome triplication, which was more than 35 % of lost genes in the *Brassica* lineage via a deletion mechanism [24]. There might be a possibility that functionally redundant *LPP* copies were lost after genome triplication, while some important copies remained. Theoretically, there should be ten *LPP* genes in *(B) napus*, which was originated through hybridizing between *B. oleracea* and *B. rapa*.

According to phylogenetic analysis, 11 *BnLPPs* were classified into four groups. Thus, *LPP* genes from *(A) thaliana* were classified into all four groups together with *BnLPPs, BraLPPs*, and *BoLPPs* genes (Fig. 2), suggesting the comparable evolutionary events between four closely related species. According to Fig. 2, several *BnLPPs* genes were closely associated with *BraLPPs*, and *BoLPPs*, indicating that these genes might have originated by segmental duplication [25]. These arrangements in the phylogenetic tree were further established by the gene structure analysis (Fig. 3). Furthermore, the motifs designs were also similar within the group organization (Fig. 3). Particularly, group IV possesses diverse gene structures and motifs designs, signifying that *BnLPPs* have very conserved protein structures. The comparative analysis of *LPPs* between *(B) napus* and its parents revealed that the *B. napus* genome is not a simple sum of *B. oleracea* and *B. rapa*. However, it undergoes many losses and duplications after hybridization, which benefited natural selection and crop domestication [23].

Almost all *BnLPPs* were predicted to be localized in the plasma membrane except *BnLPP1C*, specifically localized in the endoplasmic reticulum. These outcomes are consistent with previous studies that all PAP must bind to the membrane as a phospholipid substrate location [26]. Further, these results were also supported by the GO enrichment analysis (Additional file 7). Based on similarities among protein sequences, *BnLPPs* were classified into four groups. Although the functional studies on plant’s *LPPs* are still lacking; thus, their function could be deduced through expression patterns. In *B. napus*, the *BnLPPs* family members can be expressed in all kinds of plant tissues, indicating that LPP is an important enzyme for plant development. However, the expression profiles of *BnLPP* genes varied in the various tissues. For example, the expression of six *BnLPPs* was higher in leaves than in other tissues (Fig. 7), which is consistent with the expression level of *AtLPP1* [13–15]. Notably, some genes showed higher expression levels in leaves, stigma, stem, roots, and stamens, suggesting that *BnLPPs* might play tissue-specific roles in the rapeseed development.

Analysis of the *cis*-acting elements indicated that *BnLPPs* might respond to different stress and hormone signaling (Fig. 5). Therefore, the expression patterns of *BnLPPs* were evaluated under drought, salinity, and cold stress. Under drought stress, nine genes were up-regulated, eight genes were up-regulated under cold stress, and six genes showed higher expression against salinity. Interestingly, a few same genes also showed higher expression under different stress conditions, e.g., *BnLPP3D* and *BnLPP3D*. On the contrary, *BnLPP4B* was down-regulated under drought and salinity but was significantly increased under cold stress. In a recent study, *AtLPP1* was induced under various stresses, including DNA damage, G-protein activation, and oxidative stress, while the *AtLPP2* gene was constitutively expressed under the same stress conditions [13]. In our study, *BnLPP2A* and *BnLPP2B*, homologous to *AtLPP2*, were induced under drought, suggesting that *BnLPPs* gain new functions during evolution.

Likewise, the *cis*-elements analysis showed that *BnLPP* genes are involved in responding to various phytohormones [Fig. 5]. Phytohormones play significant roles in regulating stress responses in plants [27]. Our results confirmed that there might be cross-talk between the hormone signaling transduction and *BnLPPs*. Therefore, we analyzed the expression profiles of 11 *BnLPPs* under ABA, IAA, GA, and KT treatment. All the *BnLPPs* responded to ABA treatment, revealing that *BnLPPs* were widely involved in ABA’s signaling pathways. Hormone treatments induced the expression of *BnLPP1A* and *BnLPP3D*. On the other hand, the expression of *BnLPP1A* and *BnLPP3D* were not influenced by hormones. Our results showed that the functional gene diversification occurred during gene duplication, and the homologous genes might have different functions. Particularly, the expression level of *BnLPP4B* was decreased under hormones, and the other genes (homologous to *BnLPP4B*) showed opposite expression trends. The expression of *AtLPP2* was decreased by ABA treatment, which participates in regulating seed germination and stomatal movements [14, 15]. In the current study, the expression of both *BnLPP2A* and *BnLPP2B* was increased by ABA, suggesting that *AtLPP2* and *BnLPP2A/2B* might have different functions ABA signaling pathways.

miRNAs, a group of single-stranded non-coding micro RNAs, have been involved in post-transcriptional gene regulation [28, 29]. Recently, numerous miRNAs have been recognized by genome-wide examination in rapeseed that corresponds to diverse environmental cues [30–33]. Hence, we identified six putative miRNAs (belonging to two families, i.e., bna-miR156 and bna-miR396) targeting three *BnLPP* genes (Fig. 6). Interestingly, both miRNAs families respond to different stress conditions and developmental tissues in numerous plant species [34–37]. To our best knowledge, for the first time, we predicted that numerous miRNAs target *LPP* genes. These predictions open new windows for future investigations at a post-transcriptional level.

## Conclusions

In the present study, we identified 11 *BnLPPs*, four *BoLPPs*, six *BraLPPs*, and four *AtLPPs* genes via genome-wide analysis. Based on similarities, these genes were clustered into four groups (Group I-Group IV). For better understandings, chromosome distribution, gene duplication, gene structure, conserved motifs, *cis*-elements, GO annotation, and miRNA prediction analysis have also been performed. Furthermore, the expression pattern in different tissues and under diverse abiotic stress and phytohormone stimuli. The results revealed that the loss and duplication events occurred during the expansion of *BnLPPs* family genes. Besides, *BnLPPs* showed differential tissue-specific, abiotic stress, and hormone-responsive expression patterns. The abiotic stress and hormone-related *cis*-elements were also identified in *BnLPPs*, appropriately explaining the induction of the *BnLPPs* by stress and hormone. For the first time, we have predicted that two miRNAs families were targeting the three *BnLPP* genes. In conclusion, the extensive data collected in the present study can be used for upcoming functional analysis of *BnLPP* genes in rapeseed growth, development, response to hormone and abiotic stresses.

## Methods

### Identification of *LPP* genes in rapeseed

To identify potential *LPP*s in rapeseed, the genome sequences of *B. napus* were obtained from the Genoscope database (https://www.genoscope.cns.fr/brassicanapus/), and a local database was constructed using NCBI-blast-2.7.1 + software. BlastP search was performed (e-value 1e-^5^) using four LPP amino acid sequences of *Arabidopsis thaliana* [AtLPP1 (*At2g01180*), AtLPP2 (*At1g15080*), AtLPP3 (*At3g02600*), and AtLPP4 (*At3g18220*)] as a query as described previously [13]. Meanwhile, HMMER 3.0 [38] was used to search LPPs with the PAP2 domain (PF01569) obtained from the Pfam database [39], and the value of cutoff was 0.01. Pfam protein database (http://pfam.xfam.org/) further confirmed the PAP2 domain in candidate genes, and the redundant putative *LPPs* were excluded manually. Eleven *BnLPP* genes were finally identified in the rapeseed genome. The information of sequence lengths, molecular weights, isoelectric points, and predicted subcellular location was obtained from the ExPasy website (http://web.expasy.org/protparam/) [40].

In addition, to reveal the evolutionary relationships of *LPP* genes in different plant species, potential *LPP* genes from two plant species (*B. oleracea* and *B. rapa*) were also identified using the same method as described above. The genome sequences of the *B. oleracea* and *B. rapa* were downloaded from the JGI Phytozome v12.1 [41] database.

### Sequence analysis and structural characterization

Subcellular localization of BnLPP proteins was predicted using the WoLF PSORT server [42]. Multiple Expectation Maximization for Motif Elicitation (MEME) was used to analyze the conserved *B. napus* and *A. thaliana* protein sequences [43]. For conserved motifs discovery, the maximum number of motifs was set to 12, the classic mode and others default parameters were selected. Tbtools (V 1.068) software was used to display the *BnLPP*s gene structures [44].

### Phylogenetic analysis

MapChart V2.1 [45] was used to obtain the gene chromosomal locations. Multiple Collinearity Scan toolkit (MCScanX) [46] was used to detect gene duplication events. The syntenic relationship of *BnLPPs* with other analyzed genomes was displayed by the Circos [47] and JCVI tools [48]. KaKs_Calculator 2.0 [49] was used to calculate non-synonymous (ka) and synonymous (ks) substitution of all the duplicated *BnLPPs*.

To investigate the phylogenetic relationships of *BnLPP* genes, a total of 25 LPP proteins with PAP2 domains were identified from four plant species, and their multiple sequence alignment was carried out by ClustalW [50]. MEGA X was used to construct a phylogenetic tree via the neighbor-joining method with 1000 bootstrap replicates [51]. For a better image, the tree was displayed via EvoVIEW 2.0 [52].

### Promoter sequence analysis

The sequences of 2 Kb upstream from the start codon were downloaded from Genoscope and defined as the promoter of each *BnLPPs*. Then, PlantCARE [53] was used to predict *cis*-elements, and displayed using Tbtools [44].

### Prediction of putative miRNA targeting *BnLPP* genes and functional annotation analysis

In the current study, the *BnLPPs* CDS sequences were used to predict targeted miRNAs using the psRNATarget server (http://plantgrn.noble.org/psRNATarget/home) with default parameters, except maximum expectation (E) = 5.0. We selected the targeted sites with high degrees of complementarity shown as shown in Fig. 6. Cytoscape (V3.8.2, https://cytoscape.org/download.html) software was used to create the interaction network between the prophesied miRNAs and the equivalent target *BnLPP* genes.

For functional annotation analysis, the BnLPP protein sequences were submitted to the eggNOG website (http://eggnog-mapper.embl.de/) for GO annotation with the set “Orthology restrictions” as “Transfer annotations from one-to-one orthology only”, and other default parameters.

### Plant materials and treatments

In the present study, Westar, a rapeseed variety, was provided by the Oil Crops Research Institute of the Chinese Academy of Agricultural Sciences (CAAS), China, and was used for stress treatments. Healthy seeds were selected and were sterilized with a 10 % hypochlorous acid solution for 5 min. The seeds were germinated in a chamber (25 ℃, day/night, 16 h/8 h light/dark cycle) until the radicle’s length was reached 5 mm. These germinated seeds were used for the stress treatments. For salinity and drought treatments, the seeds were subjected to 150 mM NaCl and 15 % PEG-6000 solution on water-saturated filter paper, respectively. For cold stress, the seeds were subjected to 4 ℃ on water-saturated filter paper. For phytohormone treatment, the seeds were cultured in Murashige and Skoog (MS) nutrient solution with 100 µM abscisic acid (ABA), gibberellin (GA), kinetin (KT), and indole-3-acetic acid (IAA). The samples were harvested after 0 (CK), 2, 4, 6, 8, and 12 h of the treatments and were stored at − 80 ℃ for subsequent analysis.

The seeds were germinated as described above. They were then transplanted into nutritious soil in plant growth chambers at a temperature of 22 °C, a light intensity of 3,000 lx, and a photoperiod of 16 h light/8 h dark. Different tissues such as root, stem, leaf, flower, petal, stamen, stigma, and silique were harvested from the plants. All samples were frozen in liquid nitrogen and stored at − 80 °C until total RNA extraction and qRT-PCR analysis.

### RNA isolation and quantitative real-time PCR

RNA extraction and the cDNA synthesis were carried out using TransZol Up Plus RNA and cDNA Synthesis SuperMix (TransGen Biotech, China) according to manufacturer instructions, respectively. The qRT-PCR was carried out in ABI StepOne (Applied Biosystems, America) using the SYBR Green Supermix. *β-actin* was used as an internal control, and all the primers used in the present study are listed in Additional file 1. The qRT-PCR procedure was performed as follows: 94 °C for 10 min, followed by 40 cycles of 94 °C for 15 s, 60 °C for 30 s, 72 °C for 10 s. Each qRT-PCR reaction was carried out with three biological triplicates, and the data were examined using the 2^−△△CT^ method as described previously [54, 55]. For tissue-specific expression profiling, we followed the same method as described earlier.

## Supplementary Information

**Additional file 1: Table S1.** Sequences of the primers used in this study.

**Additional file 2: Table S2.** The protein sequences of the LPP gene family in *(A) thaliana, (B) oleracea, B. rapa*, and *B. napus*.

**Additional file 3: Table S3.** Analysis and distribution of conserved motifs in BnLPP proteins.

**Additional file 4: Table S4.** Segmentally and tandemly duplication and one-to-one orthologues relationships between *B. napus, B. rapa, B. oleracea*, and *A. thaliana.*

**Additional file 5: Table S5.** Ka/Ks analysis of *BnLPP* gene pairs.

**Additional file 6: Table S6.***Cis*-elements in the promoters of putative *BnLPP* genes.

**Additional file 7: Table S7.** The GO enrichment analysis of *BnLPP* genes.

**Additional file 8: Table S8.** The prediction of miRNAs target sites.

## Data Availability

The datasets used and/or analyzed during the current study are available from the corresponding author on reasonable request. However, most of the data is available in additional files. The sequences of *Brassica napus*, *Brassica oleracea*, and *Brassica rapa* are available in the GENOSCOPE database (https://wwwdev.genoscope.cns.fr/brassicanapus/data/) and Phytozome v12.1 (https://phytozome.jgi.doe.gov/pz/portal.html##).

## Abbreviations

LPPs: Lipid phosphate phosphatases
PAP: Phosphatidic acid phosphatase
ABA: Abscisic acid
GA: Gibberellin
IAA: Indole-3-acetic acid
KT: Kinetin
GO: Gene ontology
